# In Vitro Bone Differentiation of 3D Microsphere from Dental Pulp-Mesenchymal Stem Cells

**DOI:** 10.3390/bioengineering10050571

**Published:** 2023-05-10

**Authors:** Iñigo Gaitán-Salvatella, Patricia González-Alva, Juan José Montesinos, Marco Antonio Alvarez-Perez

**Affiliations:** 1 Tissue Bioengineering Laboratory, Postgraduate Studies, Research Division, Faculty of Dentistry, National Autonomous University of Mexico (UNAM), Coyoacán, Mexico City 04510, Mexico; igaitansalvatella@gmail.com (I.G.-S.); goap.unam@gmail.com (P.G.-A.); 2 Mesenchymal Stem Cells Laboratory, Oncology Research Unit, Oncology Hospital, National Medical Center (IMSS), POST, Mexico City 06720, Mexico; montesinosster@gmail.com

**Keywords:** 3D cell culture, cellular spheroid, biocompatibility, alp activity, bone tissue engineering

## Abstract

Bone defects lead to the structural loss of normal architecture, and those in the field of bone tissue engineering are searching for new alternatives to aid bone regeneration. Dental pulp-mesenchymal stem cells (DP-MSC) could provide a promising alternative to repair bone defects, principally due to their multipotency and capacity to fabricate three-dimensional (3D) spheroids. The present study aimed to characterize the 3D DP-MSC microsphere and the osteogenic differentiation capacity potential cultured by a magnetic levitation system. To achieve this, the 3D DP-MSC microsphere was grown for 7, 14, and 21 days in an osteoinductive medium and compared to 3D human fetal osteoblast (hFOB) microspheres by examining the morphology, proliferation, osteogenesis, and colonization onto PLA fiber spun membrane. Our results showed good cell viability for both 3D microspheres with an average diameter of 350 μm. The osteogenesis examination of the 3D DP-MSC microsphere revealed the lineage commitment, such as the hFOB microsphere, as evidenced by ALP activity, the calcium content, and the expression of osteoblastic markers. Finally, the evaluation of the surface colonization exhibited similar patterns of cell-spreading over the fibrillar membrane. Our study demonstrated the feasibility of forming a 3D DP-MSC microsphere structure and the cell-behavior response as a strategy for the applications of bone tissue guiding.

## 1. Introduction

Bone/periodontal defects caused by trauma or diseases increase each year, causing major health problems when the defects exceed the self-regenerative capacities of the tissues. The current therapies are not sufficient for solving this problem, and the need to look for new strategies to aid in regenerating bone tissue is challenging [1,2].

One strategy that has been used in regenerative medicine is the use of mesenchymal stem cells (MSC) because they represent a heterogeneous population of undifferentiated cells capable of self-renewal and multipotentiality [3]. MSC have been isolated from many different tissues, including bone marrow, adipose, cartilage, muscle, umbilical cord, and placenta [4,5,6]. Although mesenchymal cells are considered an accessible source, there are some disadvantages, such as invasive surgery, sample processing, and donor search [7].

To avoid the aforementioned drawbacks, the search for novel options for this type of cell has turned to the oral cavity, where there are sources of MSC, such as those isolated from the periodontal ligament, gum, pulp, and dental follicles, and all of the latter are accessible without costly or invasive surgeries [8,9,10,11]. However, among those that have gained relevance in bone tissue engineering are dental pulp mesenchymal stem cells (DP-MSC) because they are undifferentiated, derived from the neural crest, have fibroblastic morphology, adhere and proliferate on culture plates, are positive for CD29, CD90, CD13, CD166, CD105, CD73, CD44, and STRO-1, and negative for CD14, CD34, and CD45, and show multipotency, including osteogenic, chondrogenic, adipogenic and neurogenic capacity [12,13,14,15].

Another important aspect to consider in MSC cell-based studies is the method employed for the cell culture, in that the differentiation potential must be preserved [16]. Recently, three-dimensional (3D) culture strategies have gained relevance because they are more related to how they are found in their native microenvironment. It is proposed that they maintained the native phenotype and acquired a morphology more related to its physiological environment, favoring survival, gene expression, and differentiation potential [17].

Within 3D cultures, what is sought is simplicity, reproducibility, and scalability, all of which allow for the facilitation of interaction and increase in cell aggregation, such as those based on low-adherence plates and rotational and suspension systems [18,19]. However, in relation to the time of formation and maintenance of cells suspended without requiring a specialized medium, a magnetic levitation system has been employed to promote fast cellular aggregation [20].

Magnetic levitation is considered a guided self-assembly technique. The technique is based on the interaction of a non-toxic bioinorganic hydrogel of iron oxide and gold nanoparticles with the cells, permitting their manipulation through the use of magnets. Due to its magnetic force, this system has facilitated the aggregation to form a 3D cellular structure and co-cultures of different cell lines. The 3D cellular spheroids showed good proliferation and protein expression similar to that in vivo and had clear differences when compared to 2D traditional culture, rendering it suitable for high-throughput screening studies [21,22,23,24].

Therefore, this study aimed to report the characterization of DP-MSC microsphere formation, the osteogenic differentiation capacity, and the colonization onto fiber spun mats cultured by the magnetic levitation system and compared to 3D human fetal osteoblast microsphere in order to provide an assessment of their suitability for fabricating 3D cellular structures for bone tissue purposes.

## 2. Materials and Methods

### 2.1. Microsphere Formation and Viability Assay

The cell line used in this study was human fetal osteoblast cells (hFOB, 1.19 ATCC CRL-11372), as well as the human dental pulp mesenchymal stem cell line previously reported [11].

The 3D DP-MSC and 3D hFOB microsphere were fabricated using a magnetic nanoparticle solution (Nano Shuttle^TM^-PL; Greiner Bio-One, Houston, TX, USA) in accordance with the manufacturer’s instruction and as previously reported [22,25]. The 3D DP-MSC and 3D hFOB microsphere cultures were incubated with an osteoinduction medium cocktail (50 μM of ascorbic acid, 10 mM of β-glycerol phosphate, and 10^−7^ M of dexamethasone), which was maintained for 7, 14, and 21 days under the magnetic levitation system by the Bio-assembler Kit (Nano3D Biosciences, Houston, TX, USA). A resazurin assay was performed to quantify the cell viability on both 3D microspheres. Briefly, 10 μL of resazurin solution was added, and after 6 h of incubation, 100 μL of the medium was read at 570 nm (ChroMate, AWARNESS, Palm, FL, USA).

The morphology and diameter of the 3D DP-MSC and 3D hFOB microsphere were analyzed via fluorescence microscopy through incubation with CellTracker Green CMFDA (Invitrogen, Waltham, MA, USA) according to the manufacturer’s protocol. After 3D microspheres were observed utilizing inverted epifluorescence microscopy (AE31E model, MOTIC, Schertz, TX, USA). All microspheres were counterstained with DAPI for the nucleus. The diameter of the microsphere was obtained from the average of joining two points of the microsphere imaging and passing through its center. The experiments were conducted in triplicate and the medium was changed every 2 days.

### 2.2. Osteogenic Assay of 3D Microspheres

The osteogenic differentiation of the 3D DP-MSC and 3D hFOB microspheres was evaluated by ALP activity, calcium deposition by Alizarin Red staining (ARS, SIGMA, Livonia, MI, USA), and the expression of osteoblast-related markers by qPCR.

The intracellular ALP activity of the 3D microspheres was determined by an ALP assay kit (ABCAM, Cambridge, UK) according to the manufacturer’s protocol. Briefly, on the day of the test (days 7, 14, and 21), the microsphere was lysed and incubated with 50 μL of 5 mM of p-nitrophenol phosphate solution (pNP), and the absorbance was read at 405 nm after 1 h of incubation.

Extracellular mineralization related to calcium deposits was detected by employing an osteogenesis quantification kit based on alizarin red staining (Millipore, Burlington, VT, USA). Representative images of the 3D microsphere were captured after 21 days with a light microscope. For quantitative analysis, the alizarin red staining of both 3D microspheres was extracted by 10% of acetic acid and the eluted concentration was correlated to a standard curve of known ARS dye concentrations.

The transcriptional level of bone-related genes (RUNX2, OCN, COL1, and ALP) were quantified by using qPCR. The 3D DP-MSC and 3D hFOB microsphere were collected after 14 days for total RNA isolation by TRIzol (Invitrogen). The cDNA was synthesized using the Improm-IIReverse Transcription system and amplified by employing the appropriated primers (Table 1) and GAPDH as the control gene, utilizing the Forget-Me-Not EvaGreen qPCR Master Mix kit. The qPCR reaction was performed using the MyGoPro real-time PCR system.

### 2.3. Microsphere Colonization of PLA Fiber-Spun Membrane

Fiber-spun membranes of PLA 10% (*w*/*v*) were synthesized by air-jet spinning, as reported previously [26], onto the 12 mm cover glass. Previously, to seed the 3D microspheres, the fiber membrane was sterilized by immersion in ethanol/antibiotic solution for 30 min and air-dried under the flow cabinet. For evaluating the colonization, after 24 h of microsphere formation by a magnetic levitation technique, two microspheres of DP-MSC and hFOB were seeded in the center of the fiber-spun membrane and incubated for 3 and 7 days. At the prescribed time, the evaluation of the interaction of the 3D microsphere above the surface was evaluated by a Live/Dead Cell Imagining kit (Invitrogen) following the manufacturer’s instructions and was observed under an epifluorescence microscope (model AE31E; MOTIC, Schertz, TX, USA). DAPI was used for observing the nucleus of the cells. The experiments were conducted in triplicate.

### 2.4. Statistical Analysis

The results were expressed as the mean ± standard deviation (SD). For comparison between groups, Student’s *t*-test was employed to assess statistical significance with a *p*-value of <0.05. This was performed using SigmaPlot version 12.0 statistical software. Asterisk (*) indicates significant differences between conditions.

## 3. Results

### 3.1. Microsphere Viability

In order to assess the suitability as a facile methodology to fabricate 3D microsphere structures by a magnetic levitation system, the cell line derived from dental pulp mesenchymal stem cells (hDP-MSC) was used as a model to study the osteogenic differentiation capacity. Moreover, to assess whether the interaction of incubating hDP-MSC cells with the nanoshuttle magnetic nanoparticle solution to induce 3D microsphere aggregation under the magnetic system affects cell viability, we carried out a comparison with the human fetal osteoblasts (hFOB) cell line utilizing the resazurin assay.

**Figure 1:**
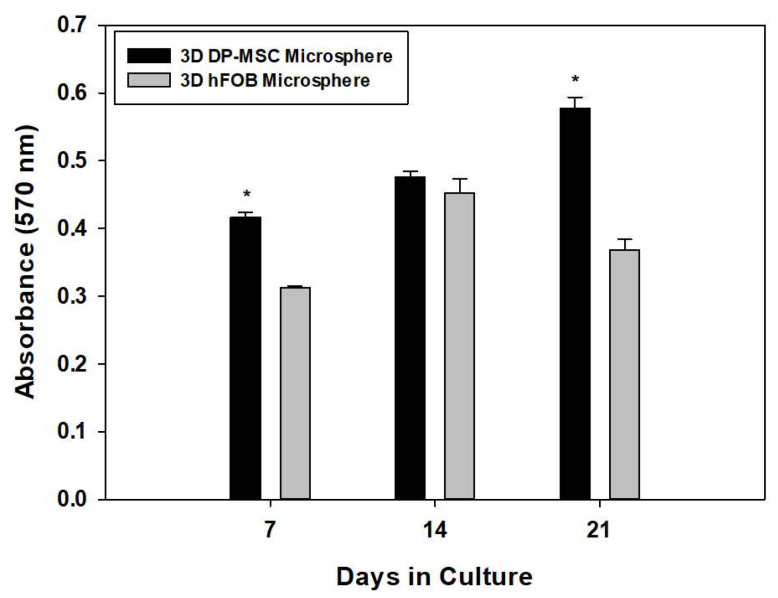
Comparison of cell viability between the 3D DP-MSC microsphere against the 3D hFOB microsphere after growth in an osteoinductive medium after 7, 14, and 21 days. This was determined by the resazurin assay. (*) Asterisk indicates significant differences between conditions a *p*-value of <0.05.

As shown in Figure 1, differences in the viability of both microspheres were observed during the time of culture. After day 7, the 3D hFOB microsphere exhibited less viability compared to the 3D DP-MSC microsphere. However, on day 14, the viability was similar to that of the DP-MSC microsphere, and on day 21, the viability was again lower than that of the 3D DP-MSC microsphere. This behavior of the cell viability could be explained by the presence of the osteogenic medium and is related to the fact that the 3D hFOB microsphere was more easily induced and committed more easily to the osteogenic phenotype than the 3D DP-MSC microsphere. However, our results demonstrated that, despite performing the same technique for the microsphere formation for hFOB and DP-MSC, both cell lines revealed good cell viability and cell-growth potential, considering they were under a differentiating stimulus toward osteogenic lineage.

### 3.2. Microsphere Morphology

**Figure 2:**
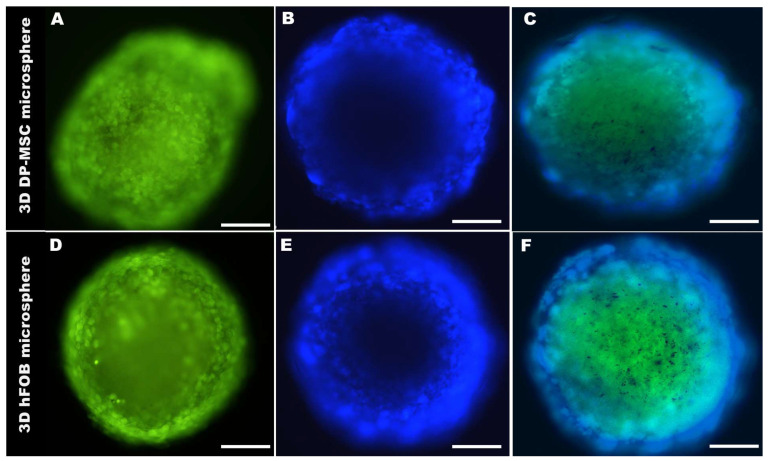
A representative micrograph of the size and morphological comparison of the 3D DP-MSC microsphere (**A**–**C**) and the 3D hFOB microsphere (**D**–**F**) after 21 days of culture. (**A**,**D**) Green marker of Cell-Tracker, (**B**,**E**) Cell nuclear staining by DAPI, and (**C**,**F**) Microsphere merge. Scale bar = 100 μm.

The morphology of the 3D DP-MSC and 3D hFOB microspheres were analyzed by fluorescently labeled cells after 21 days of culture (Figure 2). After the green staining, the cells were viable and allowed to self-assemble into an organized microsphere, in which cell–cell interaction could be visualized (Figure 2A,D), and the DAPI staining allowed for the appreciation of the nucleus in the microsphere in both cell lines (Figure 2B,E). Additionally, the low intensity of the DAPI dye in the center of the microspheres confirmed the surrender of the hypoxic center by a quiescent and proliferative layer, consistent with 3D cultures. Moreover, the merge of the 3D microsphere for DP-MSC (Figure 2C) and hFOB (Figure 2F) revealed a uniform average size with an increase in culture time, as seen in the diameter calculations listed in Table 2.

### 3.3. Osteogenic Evaluation of Microspheres

#### 3.3.1. Microsphere ALP Activity

**Figure 3:**
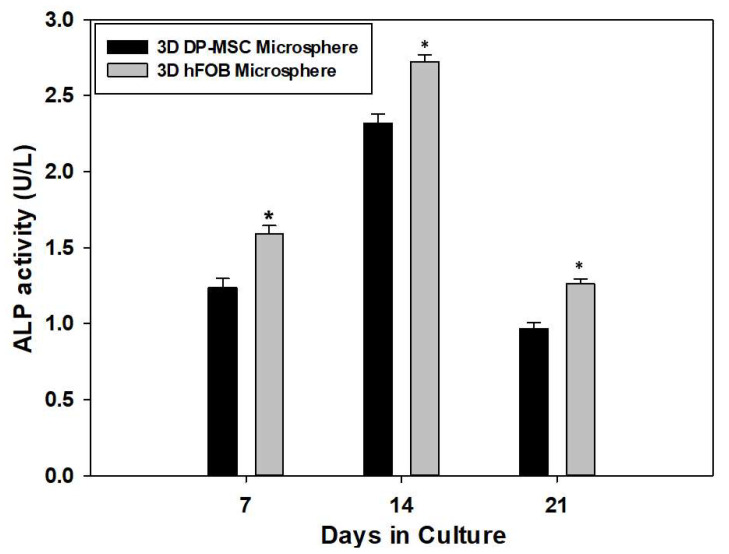
Quantitative determination of the enzymatic activity of alkaline phosphates onto the 3D DP-MSC microsphere and the 3D hFOB microsphere grown in the osteoinductive medium after 7, 14, and 21 days. (*) Asterisk indicates significant differences between conditions a *p*-value of <0.05.

The quantification of the intracellular presence of the ALP enzyme as an early marker for osteogenic differentiation is presented in Figure 3. The results reveal that ALP activity increased from day 7 and 14, with less activity on day 21 in both 3D microsphere types. Moreover, differences in the enzymatic activity during the entire culture time could be observed, wherein the 3D hFOB microsphere demonstrated more conversion of the pNP solution in comparison with the 3D DP-MSC microsphere. However, these results indicate that the microsphere can differentiate by obtaining osteogenic features during the process of bone biomineralization.

#### 3.3.2. Microsphere Extracellular Mineralization Assay

**Figure 4:**
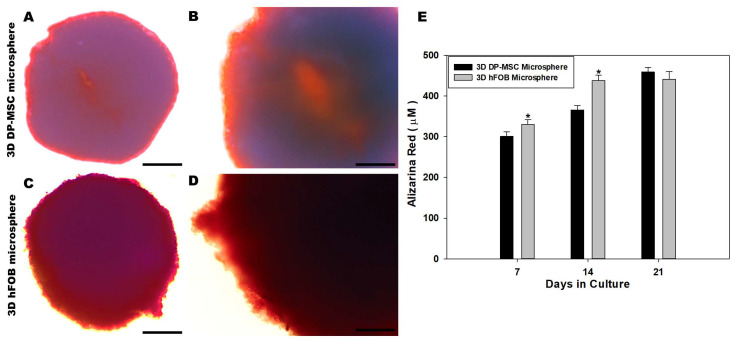
Qualitative and quantitative evaluation of the calcium deposit on the 3D DP-MSC and 3D hFOB microsphere determined by alizarin red staining. Representative microscopic view of red positive cells on the periphery and dark red toward the center of the 3D DP-MSC (**A**,**B**) and 3D hFOB (**C**,**D**) microspheres after being grown in the osteoinductive medium for 21 days. Scale bar = 100 μm. (**E**) Comparison of the eluted red staining between the 3D DP-MSC and 3D hFOB microspheres after 7, 14, and 21 days. (*) Asterisk indicates significant differences between conditions a *p*-value of <0.05.

Extracellular matrix mineralization of the 3D DP-MSC microsphere and the 3D hFOB microsphere was analyzed using Alizarin Red staining after 7, 14, and 21 days (Figure 4). Color staining related to the quantification of the eluted red positive signal was higher in the 3D hFOB microsphere than in the 3D DP-MSC microsphere after 7 and 14 days; however, at 21 days, the content revealed an increment in the 3D DP-MSC microsphere, indicating that these microspheres exhibit a late period of matrix maturation (Figure 4E). Moreover, a representative micrograph of the 3D DP-MSC and 3D hFOB microspheres after 21 days exhibited the red positive signal on the periphery of the microspheres with more intensity in the 3D hFOB microsphere (Figure 4C,D) in comparison to the 3D DP-MSC microsphere (Figure 4A,B). Nevertheless, in both microspheres, the enhanced alizarin red staining tended to increase toward the center of the microsphere.

#### 3.3.3. qPCR

**Figure 5:**
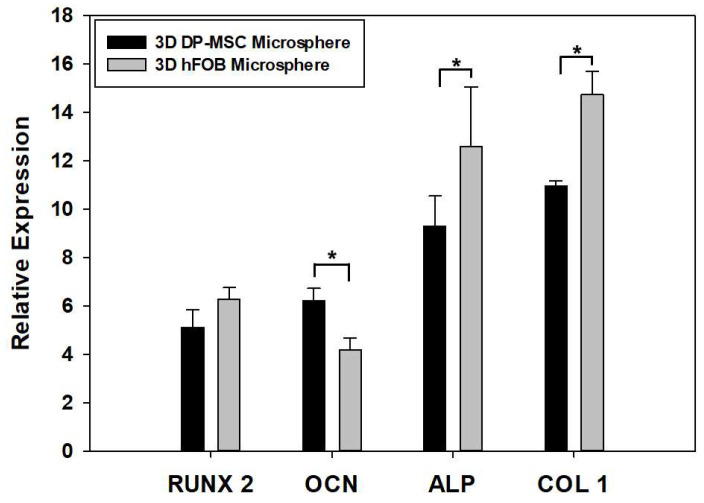
Comparison of the expression of osteoblast-related genes such as RUNX2, OCN, ALP, and Col1 between the 3D DP-MSC and 3D hFOB microspheres after 14 days in the osteoinductive medium. (*) Asterisk indicates significant differences between conditions a *p*-value of <0.05.

The qPCR analysis of osteoblast-related genes in the 3D DP-MSC and 3D hFOB microspheres such as RUNX-2, OCN, ALP, and Col1 was evaluated after 14 days of culture (Figure 5). Regarding the comparison between the gene expression on the microspheres, it was observed that there were no significant differences in the presence of the RUNX-2 gene. This is in contrast to the significant differences in the expression of ALP and Col1 in the 3D hFOB microsphere in comparison with the 3D DP-MSC microsphere, where the differences in upregulation relates to the OCN gene. However, detecting the expression of the osteogenic-related genes indicates that both microspheres revealed relative equality in the presence of mRNA toward the differentiation to the bone lineage.

### 3.4. Microsphere Colonization of the PLA Membrane

**Figure 6:**
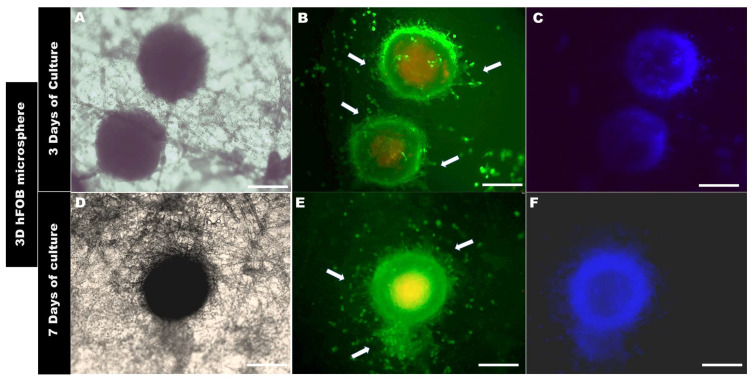
Colonization of the 3D hFOB microsphere onto the PLA fiber membrane (**A**–**C**) after 3 days and (**D**–**F**) after 7 days of culture, as analyzed by Live/Dead assay. Arrows show the cell growth zone, the colonization, and the interaction above the surface of the PLA fiber-spun mat. Scale bar = 100 μm.

**Figure 7:**
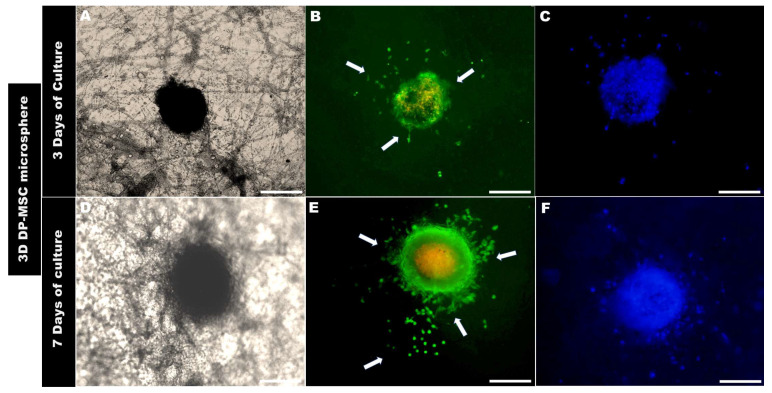
Optical micrographs of the 3D DP-MSC colonization behavior onto the PLA fiber spun membrane after (**A**–**C**) 3 days and (**D**–**F**) 7 days of culture, as evaluated by Live/Dead assay. Arrows indicate the cell’s interaction with the topographical fiber of the surface and the growth behavior above the surface of the PLA membrane. Scale Bar = 100 μm.

The evaluation of the interaction of the 3D DP-MSC and 3D hFOB microspheres on the surface of the PLA fiber-spun membrane was carried out using live and dead staining assays after 3 and 7 days (Figure 6 and Figure 7).

The morphological analysis of the 3D microsphere after 3 days of being in contact with the surface of the PLA fiber membrane showed that 3D hFOB (Figure 6B) and 3D DP-MSC (Figure 7B) cells present on the periphery of both 3D microspheres began to migrate radially. However, it can be observed that there are some differences in both microspheres. For example, the 3D hFOB microsphere is more circular and entertains a specific colonization of the viable cells that are approximately 62.96 ± 5.04 μm from the periphery of the microsphere, and the interaction of the cells with the random fibrillar topographical cue can be observed in great detail (Figure 6A–C). This is in contrast to the 3D DP-MSC microsphere, where the cells appear scattered following the orientation of the fibers with a spreading of approximately around of 75.10 ± 8.60 μm of surface colonization (Figure 7A–C).

On the other hand, the 3D hFOB microsphere, after 7 days of cultivation, exhibits an increase in the colonization of the surface of the PLA fiber membrane (Figure 6D–F). In the micrographs, a zone of high growth in the topographical surface membrane from the periphery of the microsphere of around 91.28 ± 2.38 μm could be observed, and the growth occurred in a guided manner due to the presence of the polymeric fibers around the microsphere, allowing for greater interactions amongst the viable cells that are exiting the growth zone (Figure 6E,F).

A similar behavior could be observed in the 3D DP-MSC microsphere, whereby the cells that move away from the periphery remain viable and colonize the surface topography of the membrane at approximately 193.84 ± 7.22 μm (Figure 7D–F). Moreover, the growth and colonization of the cells continues to occur radially with specific points of greater covering and interactions with the random orientation of the fiber surface of the PLA membrane (Figure 7E,F).

In our results, it is possible to observe the core center related to the senescent and dead cells associated with the limited diffusion of oxygen and nutrients, decreasing cell viability in the inner layers; however, this reduction in the viability does not affect the growth, interaction, and colonization behavior of the cells on the topographical surface of the fibrillar PLA membrane. Moreover, in the micrographs, some zones of the internal structure related to the core center are not completely stained when using DAPI for nuclear localization. A possible explanation could be related to the poor penetration of DAPI staining in the microsphere. This penetration depth can be affected depending on various factors such as the size of the microsphere, the density and compaction of the cell layers, the incubation period used for the labeling, the concentration of the dye, and due to the light-scattering in thick specimens as the microsphere. In addition, we used epifluorescent microscopy as a strategy for end-point fluorescence images of fixed microspheres that limited the analysis. A possible strategy to solve this limitation could be the microsphere clearing technique that enables 3D volumetric imaging by reducing light-scattering and improving the penetration of label dye staining [27,28].

## 4. Discussion

Bones perform important functions in life, such as providing mechanical support for locomotion, protecting organs, and controlling mineral homeostasis. These functional properties are affected by diseases or traumas that cause bone defects so that the bone itself cannot be regenerated. To solve this issue of diminished capacity induced by bone defects, bone tissue engineering is seeking new alternatives to achieve or help the regeneration of bone tissue, and one of those strategies is through the use of mesenchymal stem cells (MSC).

One of the advantages of using MSC cells is that their multipotency can help to regenerate bone defects through differentiation into bone-forming cells called osteoblasts, which are responsible for secreting, forming, and mineralizing the bone matrix. Thus, the successful use of MSC depends on the delivery method for the reconstruction of bone tissue, and the most common of these is through the use of biomaterials in order to provide an ideal microenvironment for harnessing the ability to form immature osteoblasts [29].

Recently, an alternative method related to the formation of 3D spheroid culture models has been introduced as a strategy for local delivery during the process of regenerating bone tissue. This strategy has advantages over the monolayer cultures used to seed onto the surface of biomaterials because its three-dimensionality can provide a microenvironment that mimics the interactions that take place between cells and the extracellular matrix as closely as possible to what occurs naturally [30].

In our study, we present a set of experiments to support the concept that three-dimensionality enhanced the osteogenic potential of MSC. For this, we formed a 3D microsphere using dental pulp mesenchymal stem cells that were under the stimulation of biomineralization-inducing media and cultured using a magnetic levitation system. Our data are in agreement with previous studies that have reported that the use of magnetic nanoparticles to manipulate cell aggregation by magnetic force does not cause any toxicity effects [22,31,32,33].

Likewise, our data revealed that the 3D microspheres of both cell lines present good cell viability throughout the culture time and that this may be related to the microenvironment that is favored by the 3D microspheres, due to the cells interacting more and increased signaling, as has been reported via cell–cell and cell-ECM [18,34]. Moreover, the 3D microenvironment improved the cellular plasticity, and, in our study, this could be related to better cell viability and cell growth in a 3D DP-MSC microsphere than in that of a 3D hFOB microsphere, demonstrating great advantages when working with undifferentiated cells such as DP-MSC. Indeed, the advantage of using a 3D DP-MSC microsphere is that it can promote cell-to-cell interaction and communication, enhancing their regenerative potential, and improving the survival and engraftment of transplanted cells because the microsphere could create a protective microenvironment for cells, shielding them from harsh conditions in the host tissue and allowing them to survive thanks to their immunomodulatory properties, potentially reducing the risk of rejection and improving the success of tissue regeneration [11,35,36].

In tissue engineering, 3D spheroids have become increasingly important as biofabrication models; however, their application, efficiency, or improvement in creating a 3D structure with highly uniform size, shape (geometry), and morphology presents a challenge in controlling the manipulation during the experiment [37]. In our study, the comparison between both 3D microspheres showed uniform morphology, and the size did not change significantly throughout the 21 days of culture. This uniform size is important for fabricating tissue constructs that could mimic high cell-density tissue that results in increasing cellular interactions [38]. Moreover, the diameters of both 3D microspheres are in agreement with studies employing the same magnetic levitation system that reported diameters between 300 μm and 1 mm [25,27,39].

In bone/periodontal regeneration, it is recognized that DP-MSC possesses several immunomodulatory, paracrine, and multipotency properties that make it a candidate for exploring its differentiation toward osteogenic lineage [40].

In our study, we compared the commitment toward bone differentiation of the 3D DP-MSC microsphere against the 3D hFOB microsphere under osteogenic induction medium by calcium deposition through red alizarin staining, ALP activity, and determined gene expression profiles via qPCR analysis.

Our results showed that Col1 and ALP increase in hFOB compared to DP-MSC, while RUNX2 was comparable among groups. RUNX 2 is a key transcription factor involved in the early phase of osteogenesis and remodeling, whereas ALP and COL1 are reported to increase prior to the onset of mineralization [41,42]. The process of differentiation is a well-regulated temporal sequence in which RUNX2 is essential for committing to the osteoblast lineage, provided that both 3D microsphere systems have similar expressions of the transcription factor, with no significant statistical difference. Hence, both systems were confirmed as having the same osteogenic potential supported by the ALP activity and alizarin red staining.

As for the OCN gene, our results revealed that DP-MSC has more upregulated expression, and this confirmed the commitment toward osteoblastic lineage because OCN is considered a marker involved in bone formation and the mineralization of the extracellular matrix. The differences in expression could be related to the proposed function as a late-stage marker of mineralization, which is consistent with our result, where the 3D hFOB microsphere enters more rapidly into the differentiation and mineralization stimulus than the 3D DP-MSC microsphere, supporting a more mineral deposition via alizarin red staining in the osteoblast microsphere. Moreover, the OCN upregulation in DP-MSC is considered a marker of osteoblast commitment because its expression is a marker of early osteoblast differentiation and is involved in the signaling pathways that regulate differentiation and maturation [42,43].

Our data support that the 3D microspheres in both lines possess a great capacity to respond to osteogenic induction under magnetic levitation system conditions. This typical pattern related to bone differentiation has been reported as being regulated by multiple factors and signaling pathways [44]. This signaling could be induced by the cocktail present in the osteogenic medium; however, it has also been reported that magnetic nanoparticles could regulate the differentiation by mechanotransduction stimulation and by stimulating biochemical signals [45].

Our findings demonstrate that there is a synergistic effect between the osteogenic media and the magnetic nanoparticles. This synergism could regulate cell viability, proliferation, and differentiation in both 3D microspheres and could be influenced by the 3D culture system.

Overall, the osteoblast-markers in both 3D microspheres demonstrated that the microenvironment regulates the expression of the ALP molecule, and its activity could provide a high concentration of phosphates in the earlier stages of extracellular matrix mineral deposition onto the 3D microspheres. In the continued process of biomineralization, the presence of the non-collagenous protein OCN allows for the mineralization of the ECM collagen fiber, regulating the strength and mechanical properties of the microsphere during the culture time. Moreover, the biomolecular mechanism involved in the biomineralization of the 3D microsphere requires further study.

Guide tissue engineering (GTE) is mainly based on using resorbable and non-resorbable membranes to guide the response of progenitor cells at the site of the defect in order to regenerate the tissue. One resorbable membrane that is being used in GTE is made from polylactic acid (PLA). PLA is an aliphatic polymer approved by the U.S. Federal Drug Administration (FDA) due to its excellent physical, thermal, biocompatibility, and biodegradability properties, which have rendered it ideal for use as a scaffold [46,47]. For PLA biomaterials, there is a wide variety of techniques that allow for the synthesis of scaffolds. In this study, we used air-jet spinning (AJS) for its simplicity. This technique is mainly based on the ejection of the polymer solution by gas pressure that generates the fiber with a micro- and nanometer range onto different surfaces [48,49]. Our previous studies analyzing different PLA fiber-spun membranes revealed that the scaffolds are biocompatible, permitting cell-material interaction, cell adhesion, and cell proliferation [26,50,51].

Recently, a concept has been introduced into the field of tissue engineering denominated mini-scaffolding, which seeks to combine the principles of scaffold-base and scaffold-free approaches to obtain a combinational synergy that could open a strong field toward translational medicine because it could be used as a source of cellular release at a damage site. Likewise, it is important to note that an important challenge comes in the form the cellularization of the mini-scaffold and that this could be solved by employing cell aggregates or spheroids, where it is proposed that the mini-scaffold could be seeded directly or have an entrance for the insertion of the spheroids [52].

With this idea in mind, we analyzed interaction and colonization by directly seeding the 3D DP-MSC and 3D hFOB microspheres onto the surface of the PLA fiber-spun mat in an attempt to contribute to the concept of the mini-scaffold.

From our preliminary analysis, both lines under osteogenic stimulation demonstrated a similar spreading and colonization response. This response could be regulated by the topography of the PLA membrane. The surface could be sensed through the 3D microsphere, and the cell on the periphery could be receiving the information cues to begin to spread and colonize the membrane following the random orientation of the PLA fiber. These cell behaviors are in agreement with previous studies reporting that the microenvironment, the physical signals mediated by the fibers mimicking the ECM, and the surface energy regulating the adhesive properties of the spreading and migration of cells could be utilized as a quantitative indicator of tissue biocompatibility [53,54,55].

However, at this stage of the study, we were unable to know whether the thickness of the mini-scaffold could affect the cellular response of the 3D microsphere. This is because a study that explored the responses to microfibrous scaffolds indicated that cell adhesion and proliferation could be regulated by the microstructure of the scaffold associated with the size and density of the pores [56].

It is essential to mention that 3 D microspheres are recognized for their ability to more accurately reproduce the gradients of micronutrients, including metabolites and oxygenation found in the tumor microenvironment. In this regard, flow cytometry is the standard method used to quantify the protein expression of 3D microspheres. However, the technique requires disruption of the spheroid, which results in the loss of spatial information from the microenvironment. Techniques in which 3D microspheres are embedded in hydrogel–protein hybrids, followed by clearing and denaturation, have permitted high-resolution imaging via an epifluorescent microscope [27]. In this regard, developing methods in which single-cell analyses could be performed on intact 3D spheroids is necessary.

Moreover, additional studies are necessary to understand the cellular response influenced by the PLA fiber-spun mat to generate the signal related to topotaxis and mechanotaxis, which contributes to instructing the 3D microsphere to colonize by analyzing the focal adhesion point, the cytoskeletal organization, and the evaluation of the physicochemical composition of the tissue deposited by the 3D microsphere in the in vitro model of biomineralization, as well as the application of the 3D microsphere-PLA construct in the in vivo bone calvaria defect model.

## 5. Conclusions

This study shows that DP-MSC has the potential to form a 3D microsphere through the magnetic levitation system, maintaining good morphology, viability, and differentiation ability similar to the 3D microsphere of hFOB, which is more committed to an osteogenic lineage. Moreover, the colonization and spreading properties of both 3D microspheres revealed similar behavior when cultured above the PLA fiber-spun membranes. In addition, our data suggested that 3D microsphere fabrication could be a tool and alternative strategy for the treatment of bone/periodontal defects or diseases that affect the musculoskeletal system.

## Figures and Tables

**Table 1:** Primers used for qPCR.

Name of Gene	Primers Sequence
Osteocalcin (OCN)	Forward: TGAGAGCCCTCACACTCCTC Reverse: CGCCTGGGTCTCTTCACTAC
Collagen 1 (Col 1)	Forward: GAGAGCATGACCGATGGATT Reverse: ATGTAGGCCACGCTGTTCTT
Run-related transcription factor 2 (RUNX 2)	Forward: CTCTGACCGCCTCAGTGATT Reverse: GCCTGGGGTCTGTAATCTGA
Alkaline phosphatase (ALP)	Forward: CGACCAGACGTGAATGAGAG Reverse: GCTACGAAGCTCTGCCTCCTG
Glyceraldehyde 3-phosphate dehydrogenase (GAPDH)	Forward: GCATCCTGGGCTACACTGAG Reverse: TGCTGTAGCCAAATTCGTTG

**Table 2:** Average diameter comparison of the 3D DP-MSC microsphere and the 3D hFOB microsphere grown in the osteogenic medium after 7, 14, and 21 days.

Day of Culture	3D DP-MSC Microsphere	3D hFOB Microsphere
7	310.68 ± 11.83 μm	321.56 ± 13.08 μm
14	329.99 ± 4.16 μm	342.67 ± 13.97 μm
21	427.05 ± 11.11 μm	432.09 ± 11.38 μm

## Data Availability

All data generated for this study are included in the manuscript.
